# Editorial: Using behavioral theories to improve medication use

**DOI:** 10.3389/fphar.2026.1817864

**Published:** 2026-03-11

**Authors:** Yifei Liu, Shikhar Shrestha, Amy Hai Yan Chan

**Affiliations:** 1 Division of Pharmacy Practice and Administration, University of Missouri – Kansas City School of Pharmacy, Kansas City, MO, United States; 2 Department of Public Health and Community Medicine, Tufts University School of Medicine, Boston, MA, United States; 3 School of Pharmacy, University of Auckland, Auckland, New Zealand

**Keywords:** behavior theory, health behavior, health outcome, medication management, medication use

Medication therapies are the cornerstone of modern healthcare with an irreplaceable role in managing and treating a broad range of health conditions. However, clinical effectiveness is affected not only by the pharmaceutical or pharmacological properties of medications, but also by how people interact with them within real world settings. Glanz et al. provide a comprehensive synthesis of major behavioral theories, along with practical guidance for systematically applying these frameworks in health research and intervention development (Glanz et al., 2015). In addition to the existing literature, the Research Topic “Using Behavioral Theories to Improve Medication Use” in *Frontiers in Pharmacology* brings together 13 articles that explore the application of behavioral theories to improve medication adherence, safety, and patient engagement across diverse populations and care settings. Spanning different healthcare contexts, policy changes, disease states, and behavioral frameworks, these articles demonstrate that medication use emerges from a complex interplay of individual beliefs, social norms, healthcare professionals’ interactions, and environmental factors.

Theoretical models can specify mechanisms that drive decision-making, and articulate why patients with similar demographic profiles may adopt different medication use patterns. A coherent narrative of this Research Topic is that medication use is best understood as a dynamic and complex behavioral process, depending on individuals’ belief systems, past experiences, and social influences. For example, older adults require self-efficacy to interpret and manage hypertension regimens (Lu et al.), HIV patients facing stigma have psychosocial stressors (He et al.), pediatric asthma therapies are influenced by parental knowledge, attitudes, and practices (Tang et al.), beliefs about necessity and concerns exert influence in antibiotic demand (Almeshal et al.), and self-rated health affects information search for a pharmaceutical treatment (Liu et al.).

Another prominent theme is the focus on contextual factors such as geographics, culture, access, and policy that shape the behaviors surrounding medication use. Articles in the collection highlight diverse settings, such as rural (Li et al.) vs. urban areas (Montuori et al.), ethnically and culturally diverse populations (Tezcan-Güntekin et al.; Chery et al.), varying degrees of healthcare access (Shrestha et al.), to show that medication use is shaped by broader structural and systemic factors. The inclusion of a study on collaborative intention between pharmacists and physicians (Liu et al.) acknowledges that healthcare professionals’ balancing of self-identity and subjective norm, namely how they perceive their roles and expectations within their professional context, have collaborative behavioral underpinnings. In addition, behavioral theories do more than explaining adherence, it provides the levers for change, enabling clinicians, policymakers, and healthcare systems to design interventions that resonate with real people living real lives. For instance, an organizational, hospital-based design for a pharmaceutical care model can attend to outpatients to enhance health outcomes (Zarate-Tamames et al.). Additionally, validated measures like the BMQ-AIR© enable targeted, belief-focused interventions toward medication adherence support (Foot et al., 2024).

Moving forward, researchers and practitioners should further integrate behavioral theories into the design, implementation, and evaluation of medication-related interventions. Such integration should remain responsive to diverse populations, cultural contexts, and evolving healthcare environments, and be adaptable and sensitive to the social and structural determinants that shape medication use behaviors. Bartholomew Eldredge et al. emphasize translating selecting theory-informed methods and operationalizing them into practical, contextually appropriate intervention strategies (Bartholomew Eldredge et al., 2016). Meanwhile, as suggested by this Research Topic, strengthening collaboration among healthcare professionals is equally critical. Effective communication, shared decision-making, and clearly defined interprofessional roles are foundational to optimizing health outcomes. Medication management is inherently multidisciplinary; therefore, coordinated care models that align pharmacists, physicians, nurses, and allied health professionals can reduce fragmentation and enhance adherence support in a consistent and sustainable manner. Policymakers also play a pivotal role in this process by designing reimbursement structures and quality metrics that incentivize collaborative practice and integrated care delivery. Moreover, sustained impact requires continuous evaluation by both individuals and health providers informed by validated assessment tools. Measurement of patient beliefs, knowledge, self-efficacy, or behavioral patterns enables clinicians to identify barriers to medication adherence early and adjust interventions accordingly. By embedding iterative assessment within routine care, healthcare systems can transition from static interventions to data-informed management.

In summary, this Research Topic sends a clear message: medication use is a behavioral journey co-shaped by beliefs, norms, motivations, and environments. Behavioral theories provide a roadmap not only for understanding that journey but also for designing interventions that respect human complexity while improving health outcomes. The 13 articles collectively establish the value of integrating behavioral theories into healthcare practice to optimize medication use. The evidence generated here can be used to design targeted, theory-based interventions and policy initiatives for optimizing medication use across diverse populations and care settings. More broadly, these studies illustrate how patient beliefs and interprofessional collaboration can be operationalized through various theoretical frameworks. Behavioral theories do not merely complement clinical expertise but strengthen it by clarifying action mechanisms and guiding intervention designs. Embracing a theory-based approach is therefore important to advancing equitable, effective, and sustainable improvements in medication use.
